# A Spook That Will Not Down

**Published:** 1894-07

**Authors:** 


### EDITORIAL DEPARTMENT.

F. E. DANIEL, M. D., Editor.
S. E. HUDSON, M. D., Managing Editor.
A. J. SMITH. M. D., Galveston, Associate Editor.

EDITORIAL STAFF:

PROF. J. E. THOMPSON, M. D., Texas Medical College, Galveston; Surgery.
PROF. WM. KEILLER, M. D., Texas Medical College, Galveston; Obstetrics and Gynecology.
PROF. DAVID CERNA, M. D., Texas Medical College, Galveston; Therapeutics.
PROF. A. J. SMITH, M. D., Texas Medical College, Galveston; Medicine.
DR. ISADORE DYER, Tulane University; Dermatology.
DR. R. H. L. BIBB, Saltillo, Mexico; Foreign Correspondent.
DR. ROBT. MORRIS, Charity Hospital, N. O.; Clinical Reports

### A SPOOK THAT WILL NOT DOWN.

Dr. Swearingen's paper in this issue will be read with unusual interest. It is not only an able and well written paper and worthy of its distinguished author, but it is upon a subject of great and growing interest to every medical man; one which, at this time, is receiving a large share of attention, not only at the hands of physicians, but by legal gentlemen, and especially *railroad attorneys*. This paper antagonizes, in a measure, a vast monied interest and a large share of professional opinion, and will doubtless create surprise, and in some quarters, disquiet. It is the *first gun of the campaign* in a war involving right and justice, as well as vast sums of money; a war to settle the question whether "Railroad Spine" is a "*spook*," a "myth," a "vampire," or whether it is an *entity*, a very real thing, a costly thing, that will not down.

The writer calls attention of readers to the indications that the organization of National Railway Surgeons has a deeper signification than appears on the surface. The presence at the Galveston meeting of Judge Clark Bell, of New York,—a distinguished railroad attorney—the man who drew up the first bill chartering the great Northern Pacific railway system,—the editor of the *Medico-Legal Journal* and the originator of the Section on Railway Surgery in the Medico-Legal Society of America, is significant of itself, of a deeper object than that usually actuating surgical and medical organizations; the fact that he read a paper on that occasion strongly adverse to the idea of the existence of railroad spine—and the paper itself, are both significant. And something of the underlying purpose may be inferred from his appeal to the combined strength of the Railroad Surgeons of America to make war upon, to put down, to jugulate, destroy, annihilate, exorcise or in some way get rid of this "vampire" which, he says, "is sucking the life blood" of so many corporations!

But the most significant thing yet, is the fact that every paper read at that gathering, on the subject, was in the same line; and, so far as we can now recall, every paper on the subject which has been published recently, has been directed against railroad spine, *with one exception;* that of Prof. Allen J. Smith in TEXAS MEDICAL JOURNAL—1894. This paper by Dr. Swearingen is really the first gun fired for the defense, and its echo will reverberate throughout the country awakening responses wherever it is read. We look to see it bring on a general engagement all along the lines, so to speak.

It really begins to look, as is intimated by Dr. Swearingen, as if the National Railroad Surgeons Association is to be a kind of *kindergarten for the education of witnesses for the future;* as if a systematized effort is being, and to be made to cultivate a sentiment adverse to the belief that serious injury to the spinal cord can be inflicted without external and visible lesion, so that in due time, railroad surgeons, as experts, before courts in damage suits, can honestly and conscientiously swear that there is, and can be no such thing as railroad spine; they will have been educated to it by papers and publications of the sort alluded to, and by statistics like those of Dr. Outten, and other methods yet to be developed perhaps; and it can be seen at a glance what valuable witnesses they will be for the defense.

Poor Erichsen! he has raised a ghost, which, like that of Bancho, will not down: it can not be non-suited—nor pooh poohed—nor laughed out of court,—no matter how ugly, how dangerous, nor how costly to corporations. This paper takes up the cudgel for defense; is the first shot for the much feared, much cussed and discussed "*railway spine.*" It is not, as the doctor says,—"a question of dollars and damages"—but one of science. Did Erichsen tell the truth? Is there such a thing as railway spine? It looks very much like it, and that it has come to stay.

APROPOS OF PROPAGATING WITNESSES for the defense:—how is this, coming from the *Kansas City Medical Index.* (May, 94):

Dr. Geo. Emerson, of Winfield, Kansas, was the local surgeon of the Santa Fe road, of which Dr. G. W. Hogeboon is chief surgeon. The chief complained that the drug bills on Dr. Emerson's prescriptions for railroad employes were too heavy; that drugs dispensed on his prescriptions *cost more than the average* at other points along the line; and actually ordered him to *prescribe cheaper medicines!* And that too, let it be remembered—when every employe is taxed a certain per cent. of his wages each month for the very purpose of keeping up a hospital for employes; the drugs were being paid for by the employes' own money.

Dr. Emerson, who, the *Index* says, is "one of the brightest men in the medical profession of the Sunflower State"—as a man of spirit and honor, had but one course to pursue, and he pursued it—in a rather emphatic manner—it will be seen. He remonstrated, courteously, stated that no one should dictate what he should prescribe; that he had the patients' good at heart, and did not stop to consider the cost of the drugs he thought were needed. But this was not satisfactory, and Dr. Hogeboon notified him that his resignation was in order. It was then that Dr. Emerson telegraphed. "You and the Santa Fe may go to hell!" But he refused to resign or give up his pass, very properly.

BUT THE GIST of this matter is not yet come. Chief Surgeon Hogeboon on February 5th, says the *Index*, wrote to Troup & Brown, the Santa Fe attorneys at Winfield, asking whom to appoint a local surgeon as Dr. Emerson's successor, stating that "he should be a graduate of the regular school, competent in the practice, and, as you are well aware, *one that will make a good witness*." [ ! ! ! ! phe-w!]

Taken in connection with the foregoing remarks, it begins to look very much to a man up a tree as if one's *swearing qualities* are, in future, to be taken largely into consideration when applying for appointment as local railroad surgeon; and that certain attorneys fearing, may be, that too little attention has been paid in the past to the cultivation of that feature of the average doctor's education,—are co-operating in a movement to *grow witnesses to order*, as it were; to have a crop coming on, from which to draw, to fill vacancies whenever one of Dr. Emerson's sort kicks. See?

